# MRI-Based Analysis of Intracerebral Hemorrhage in Mice Reveals Relationship between Hematoma Expansion and the Severity of Symptoms

**DOI:** 10.1371/journal.pone.0067691

**Published:** 2013-07-02

**Authors:** Hideaki Matsushita, Masanori Hijioka, Akinori Hisatsune, Yoichiro Isohama, Shigeto Iwamoto, Hiroaki Terasawa, Hiroshi Katsuki

**Affiliations:** 1 Department of Chemico-Pharmacological Sciences, Graduate School of Pharmaceutical Sciences, Kumamoto University, Kumamoto, Japan; 2 Priority Organization for Innovation and Excellence, Kumamoto University, Kumamoto, Japan; 3 Department of Structural Bioimaging, Graduate School of Pharmaceutical Sciences, Kumamoto University, Kumamoto, Japan; Massachusetts General Hospital/Harvard Medical School, United States of America

## Abstract

Intracerebral hemorrhage (ICH) is featured by poor prognosis such as high mortality rate and severe neurological dysfunction. In humans, several valuables including hematoma volume and ventricular expansion of hemorrhage are known to correlate with the extent of mortality and neurological dysfunction. However, relationship between hematoma conditions and the severity of symptoms in animal ICH models has not been clarified. Here we addressed this issue by using 7-tesla magnetic resonance imaging (MRI) on collagenase-induced ICH model in mice. We found that the mortality rate and the performance in behavioral tests did not correlate well with the volume of hematoma. In contrast, when hemorrhage invaded the internal capsule, mice exhibited high mortality and showed poor sensorimotor performance. High mortality rate and poor performance in behavioral tests were also observed when hemorrhage invaded the lateral ventricle, although worsened symptoms associated with ventricular hemorrhage were apparent only during early phase of the disease. These results clearly indicate that invasion of the internal capsule or the lateral ventricle by hematoma is a critical determinant of poor prognosis in experimental ICH model in mice as well as in human ICH patients. MRI assessment may be a powerful tool to refine investigations of pathogenic mechanisms and evaluations of drug effects in animal models of ICH.

## Introduction

Intracerebral hemorrhage (ICH) is a severe type of stroke featured by hematoma formation within brain parenchyma that occurs most frequently in the putamen, a part of dorsal striatum. The mortality rate after ICH is high, ranging from 20 to 50% [1]. In addition, patients surviving ICH incidents exhibit contralateral sensorimotor dysfunctions as a result of neuropathological changes [2]. These poor outcomes are intractable, and there is an urgent need for development of effective therapies for ICH [3], [4].

The severity of symptoms of ICH patients such as mortality rate and neurological dysfunctions has been correlated with the volume of hematoma and initial Glasgow coma scale [5]–[7]. Indeed, inhibition of hematoma expansion does not necessarily result in amelioration of ICH symptoms. For example, administration of activated recombinant factor VII (fVIIa) that could halt enlargement of hematoma after ICH failed to produce significant effects on mortality rate and neurological dysfunctions of patients in the phase III fVIIa for Acute Hemorrhagic Stroke Treatment (FAST) trial [8]. Another variable to be considered is the location of hematoma within the brain. That is, hemorrhage expansion into the ventricles has been associated with high rate of mortality in many studies [6], [7], [9]. Injury of the internal capsule (IC), the white matter area containing axonal fibers of descending upper motor neurons and ascending sensory neurons, has also been recognized as a key event leading to motor hemiparesis after ICH [10]–[12].

Animal models of ICH are valuable for experimental studies on pathogenic mechanisms and potential therapeutic interventions. Commonly utilized models are based on intrastriatal injection of autologous blood or collagenase [13]. In particular, collagenase model is thought to produce pathological features that closely resemble those associated with ICH in human patients. However, as compared to the model based on injection of a given volume of autologous blood, the extent of pathological changes of collagenase model in individual animals tends to be variable because this model relies on extravasation resulting from enzymatic rupture of vascular walls [14]. Exploration of effective drug therapies using animal model of diseases requires stability, controllability and reproducibility of the model, and therefore, the variability of the degree of pathological changes in collagenase model of ICH should be minimized as far as possible.

Magnetic resonance imaging (MRI) is a powerful tool that enables non-invasive examination of the conditions of hematoma such as the volume and the invaded area. Based on this technique, ICH models may be standardized by exclusion of animals bearing extraordinarily small or large hemorrhage, or hemorrhage produced in abnormal locations. Moreover, relationship between the conditions of hematoma and the severity of neurological symptoms can be assessed by combination of MRI and behavioral testing, but this issue has not been addressed in detail by previous studies [15]. The present study was aimed to examine whether the conditions of hematoma in some way associated with the severity of symptoms after ICH in mouse model, as in the case with human ICH patients.

## Materials and Methods

### Ethics Statement

All procedures were approved by Kumamoto University ethical committee concerning animal experiments (Permit Number: D-24–220), and animals were treated in accordance with the Guidelines of the United States National Institutes of Health regarding the care and use of animals for experimental procedures. Male C57BL/6J mice at 9 to 10 weeks of age weighing 22 to 28 *g* were used to produce collagenase-induced model of ICH. All surgery was performed under anesthesia with pentobarbital, and all efforts were made to minimize animal suffering. Animals were maintained at constant ambient temperature (22±1°C) under a 12-h light/dark cycle (lights on between 8∶00 and 20∶00), with food and water available ad libitum.

### Induction of ICH

ICH was induced by injection of collagenase into mouse striatum, essentially according to the methods described previously [16]. Briefly, mice were placed in a stereotaxic frame under anesthesia with 50 mg/kg pentobarbital. A burr hole was made on the skull and a 30-gauge needle was inserted into the striatum. In one group of animals (group 1), the needle tip was placed for hematoma to invade IC: 0.8 mm posterior and 2.3 mm lateral from the bregma, 3.5 mm below the skull. In the other group of animals (group 2), the needle tip was placed at following stereotaxic coordinates to minimize the probability of hematoma invasion of IC: 0.2 mm anterior and 2.3 mm lateral from the bregma, 3.5 mm below the skull. Type VII collagenase (Sigma, St Louis, MO, USA) at 0.025 U in 0.5 µl saline was infused at a constant rate of 0.20 µl/min with a microinfusion pump. Sham-operated mice received injection of the same volume of saline. Body temperature was maintained at 37°C during surgery. After surgery, mice were returned to their home cage where food and water were placed in positions easily accessible even for mice with impaired sensorimotor functions. The overall vital conditions of mice were monitored everyday after ICH surgery, including daily body weight changes for the first 7 days. In most cases, body weights of mice initially decreased and then recovered after 3 days if the mice survived. A small population of mice showed no recovery of body weights even after 3 days and exhibited worsened systemic conditions: in this case, humane endpoints were applied to these mice by decapitation.

### Behavioral Tests

Sensorimotor functions of mice were evaluated by beam-walking test and modified limb-placing test [16] at 6 h, 1 d, 3 d, 1 week, 2 weeks, 3 weeks and 4 weeks after induction of ICH. Mice were trained once daily for 3 d before surgery. Evaluation of motor performance was conducted by an experimenter blinded to the conditions of hematoma in each mouse. In the beam-walking test, mouse was placed on a beam (6 mm wide, 1.1 m long and 50 cm high), and fault rate during crossing the beam as well as walking distance was obtained as an averaged value from three trials on each day. Usage of hindlimb during crossing the beam was also analyzed as a performance score on the basis of an eight-point scale as described previously [16].

Modified limb-placing test consisted of two limb-placing tasks that assessed the sensorimotor integration of the forelimb and the hindlimb by testing responses to tactile and proprioceptive stimuli [16]. First, mouse was suspended 10 cm over a table, and the stretch of the forelimbs toward the table was observed and evaluated: normal stretch, 0 point; abnormal flexion, 1 point. Next, the mouse was positioned along the edge of the table, and forelimbs were placed out of the table, suspended over the edge and allowed to move freely. Each forelimb was gently pulled down, and retrieval and placement were observed. Finally, the mouse was placed toward the table edge to examine lateral placement of the forelimb. These three tasks were scored in the following manner: normal performance, 0 point; performance with a delay (2 s) and/or incomplete performance, 1 point; no performance, 2 points. Totally, 7 points means maximal neurologic deficit and 0 point means normal performance.

### Magnetic Resonance Imaging

MRI examinations were conducted at 6 h, 1 d, 3 d, 1 week and 4 weeks after induction of ICH, following behavioral tests mentioned above. Biospec 7-Tesla 70/20 USR (Bruker Biospin KK, Yokohama, Japan) with mouse brain surface coil was used [17]. Mice were anesthetized with isoflurane, and a 3-plane scout imaging sequence was used to adjust the position of the head of mouse until the central slice was located at the level of the largest area of hemorrhage. Then, T2 weighted images (turbo RARE pulse sequence, TR 3839.5 ms, TE 47.6 ms, FOV: 2.5×2.5 cm, matrix: 500×500, RARE factor 8, 25 slices, 0.5 mm thickness) were acquired. Volume and position of hematoma were estimated from these T2-weighted images using OsiriX free software. Lesioned area was extracted by outlining the regions of hypo-hyperintensity that were clearly different from the other tissues in each slice. Hematoma volume (in mm^3^) was determined by integration of the lesioned area in each section over the section depth.

### Histology

At 4 weeks after induction of ICH, mice were anesthetized with pentobarbital and perfused transcardially with 30 ml of ice-cold phosphate-buffered saline followed by 30 ml of 4% paraformaldehyde. Brains were isolated and fixed in 4% paraformaldehyde overnight and then soaked in 15% sucrose overnight at 4°C. After being frozen, they were cut into coronal sections of 30 µm in thickness. Sections around the injection site were collected, mounted onto slides, and processed for Luxol fast blue staining for myelin. Briefly, sections were incubated overnight at 58°C in 0.1% Luxol fast blue solution in 95% ethanol containing 0.05% acetic acid. After wash with 95% ethanol, sections were exposed to 0.05% lithium carbonate solution for 5–10 s, washed with ethanol and then with water, and coverslipped.

### Statistics

All data are presented as means ± S.E.M. Survival rate of mice was statistically analyzed by Log-rank test. Time course data on behavioral measurements and hematoma volume were analyzed by two-way analysis of variance followed by post hoc comparisons with Bonferroni method. Other data were analyzed by non-parametric Mann-Whitney test for two group comparisons. Correlation between the hematoma size and the risk of death was analyzed as hazard ratios with the use of a Cox proportional-hazards model, by the R software program (version 2.8.1, R Foundation for Statistical Computing, Vienna, Austria). Correlation between the hematoma size and the degree of neurological dysfunctions was analyzed by non-parametric Spearman’s rank correlation test. Two-tailed probability values less than 5% were considered significant.

## Results

### Hematoma Volume does not Correlate Well with Mortality Rate and Neurological Dysfunctions

**Figure 1:**
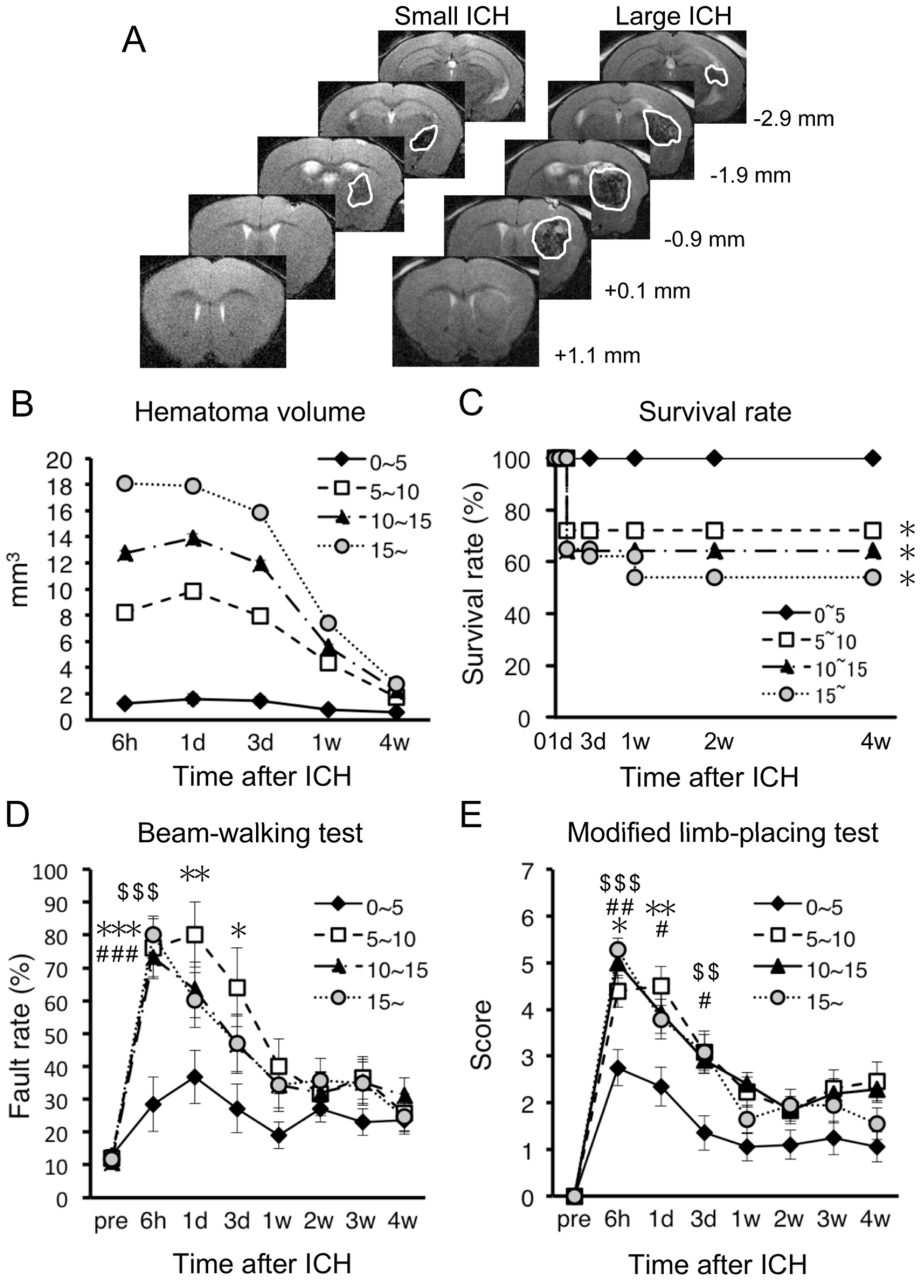
Relationship of hematoma volume with mortality and neurological dysfunctions in mouse ICH model. (A) Representative images of T2-MRI scans (+1.1 mm to −2.9 mm relative to bregma) in a mouse with small hematoma and a mouse with large hematoma at 6 h after induction of ICH. Boundary between hematoma and surrounding tissues is indicated by solid white line. (B) Time-dependent changes of hematoma volume in four groups of mice categorized by the initial volume of hematoma at 6 h after induction of ICH; <5 mm^3^ (*n* = 20 at 6 h), 5–10 mm^3^ (*n* = 18 at 6 h), 10–15 mm^3^ (*n* = 38 at 6 h) and >15 mm^3^ (*n* = 37 at 6 h). (C) Survival rate of mice after ICH. (D) Performance of mice in the beam-walking test evaluated by hindlimb fault rate. (E) Performance scores in the modified limb-placing test. Data sets consist of values derived from all mice surviving at each time point. *P<0.05, **P<0.01, ***P<0.001, 5–10 mm^3^ group versus <5 mm^3^ group; ^#^ *P*<0.05, ^##^ *P*<0.01, ^###^ *P*<0.001, 10–15 mm^3^ group versus <5 mm^3^ group; ^$$^ *P*<0.01, ^$$$^ *P*<0.001, 10–15 mm^3^ group versus <5 mm^3^ group.

In human ICH patients, large volume of hematoma has been associated with high mortality rate [5]. Accordingly, we first analyzed whether the same relationship could be applied to mouse ICH model. We used two groups of mice that received collagenase injection at different site each other: one group received collagenase injection near IC (*n* = 56), and the other group received injection at a site distant from IC (*n* = 55). In both groups, hematoma volume varied substantially among individual mice, although the same amount of collagenase solution was injected at defined stereotaxic coordinates (Fig. 1A). Irrespectively of the site of collagenase injection, we divided these mice into four groups based on initial hematoma volume (at 6 h after induction of ICH) of <5, 5–10, 10–15 and >15 mm^3^. In each group, the hematoma volume decreased gradually during 4 weeks of the observation period (Fig. 1B). The survival rate of mice with initial hematoma volume less than 5 mm^3^ was 100% after 4 weeks, whereas that of other three groups of mice ranged from 54% to 72% (Fig. 1C). There was no significant difference in the survival rate among these three groups with hematoma volume larger than 5 mm^3^. Correlation between the hematoma size and the risk of death was analyzed as hazard ratios with the use of a Cox proportional-hazards model. Among all mice, the risk of death was significantly increased with increasing hematoma size (hazard ratio = 1.1094, *p* = 0.00161). However, among the mice with hematoma size of >5 mm^3^, the risk of death did not correlate with the hematoma size (hazard ratio = 1.0493, *p* = 0.253).

We next analyzed relationship between the hematoma volume and the degree of neurological dysfunctions. In the beam-walking test, we used a beam with 6 mm width instead of a beam with 15 mm width used in previous studies [16], [18], because the narrower one could detect long-term dysfunctions of hindlimb use more reliably. The fault rate of hindlimb steps of mice with hematoma smaller than 5 mm^3^ exhibited a modest increase that did not exceed 40% during the entire course of experiments for 4 weeks. On the other hand, the other three groups of mice with hematoma larger than 5 mm^3^ (5–10, 10–15 and >15 mm^3^) exhibited a prominent increase in the hindlimb fault rate, and the values of fault rate were not apparently different among three groups (Fig. 1D). Statistical analysis with Spearman’s rank correlation test revealed that the fault rate at 6 h in the beam-walking test correlated significantly with the hematoma size when all mice were included in the analysis (*r* = 0.3342, *p* = 0.0003) but not when mice with hematoma size of >5 mm^3^ were analyzed (*r* = 0.0492, *p* = 0.6938). In the modified limb-placing test, we found a similar tendency of motor dysfunctions of each group to that observed in the beam-walking test. That is, mice with the smallest hematoma volumes (<5 mm^3^) exhibited modest level of dysfunction in forelimb use, whereas the other three groups of mice showed a substantial increase in neurological scores, which were not significantly different among three groups (Fig. 1E). Performance scores at 6 h in modified limb-placing test correlated significantly with the hematoma size when all mice were included in Spearman’s rank correlation analysis (*r* = 0.3757, *p*<0.0001), but not when only mice with hematoma size of >5 mm^3^ were analyzed (*r* = 0.1302, *p* = 0.2135). Overall, these results indicate that, when the hematoma volume exceeds 5 mm^3^, the severity of prognosis does not correlate with the absolute volume of hematoma.

### Severe Motor Dysfunctions at Early Phase Predicts High Mortality Rate

**Figure 2:**
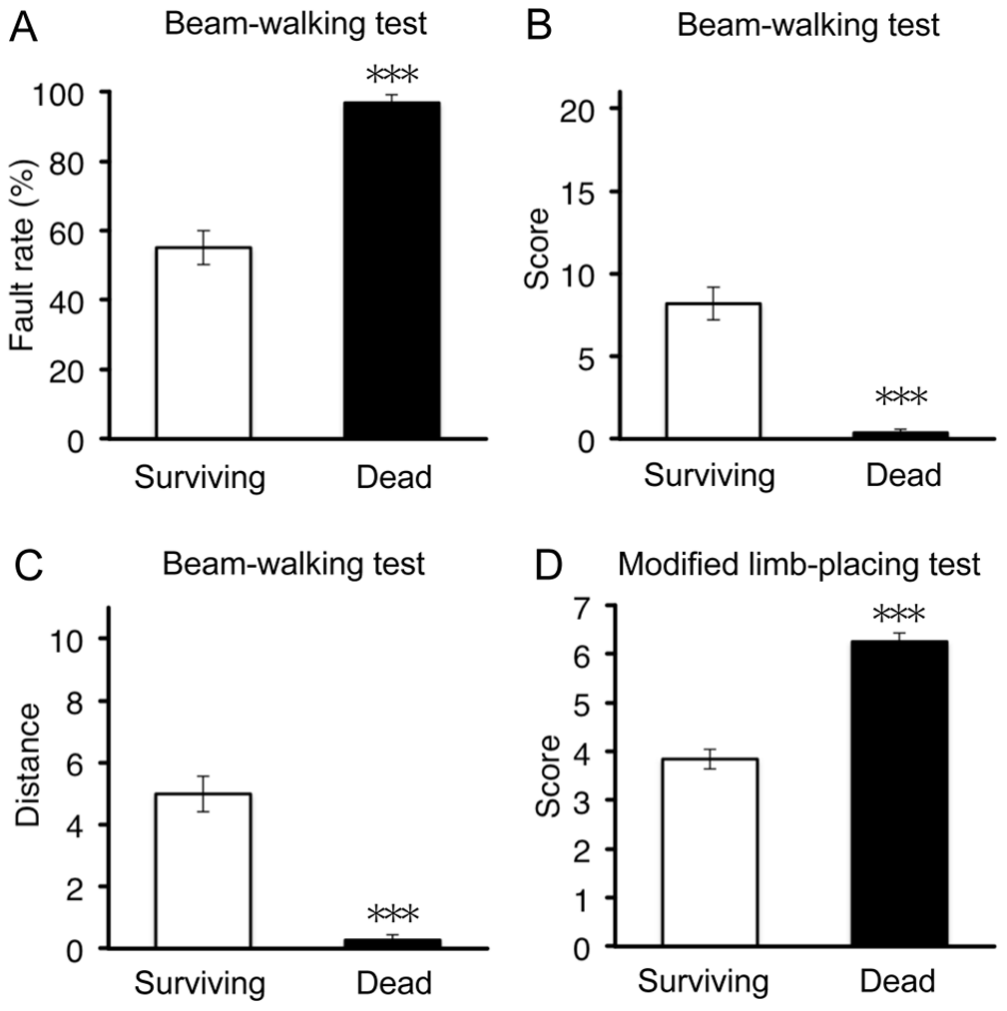
Relationship between the degree of initial neurological dysfunction and mortality of mice after ICH. Surviving mice (*n* = 78) and dead mice (*n* = 35) as of 4 weeks after induction of ICH were compared by behavioral parameters assessed at 6 h after ICH; hindlimb fault rate (A), performance score (B) and walking distance (C) in the beam-walking test, and performance score in the modified limb-placing test (D). ***P<0.001 versus dead group.

In humans, high score in Glasgow coma scale during the acute phase of ICH is a predictor of high mortality rate [6], [7]. Therefore we examined relationship between initial neurological scores and mortality of mice in ICH model. Data on neurological dysfunctions at 6 h after induction of ICH from all mice were divided into two groups, one from mice surviving 4 weeks after ICH (“surviving” group) and the other from mice dead during 4 weeks of the observation period (“dead” group). With regard to data on beam-walking test, “dead” group exhibited a higher fault rate, a lower performance score, and a shorter walking distance than “surviving” group (Fig. 2A–C). “Dead” group also showed a higher neurological score than “surviving” group in the modified limb-placing test (Fig. 2D). These results suggest that the severity of early sensorimotor dysfunctions is closely associated with mortality after experimental ICH in mice.

### Induction of Hemorrhage Near Internal Capsule Results in High Mortality Rate and Severe Neurological Dysfunctions

**Figure 3:**
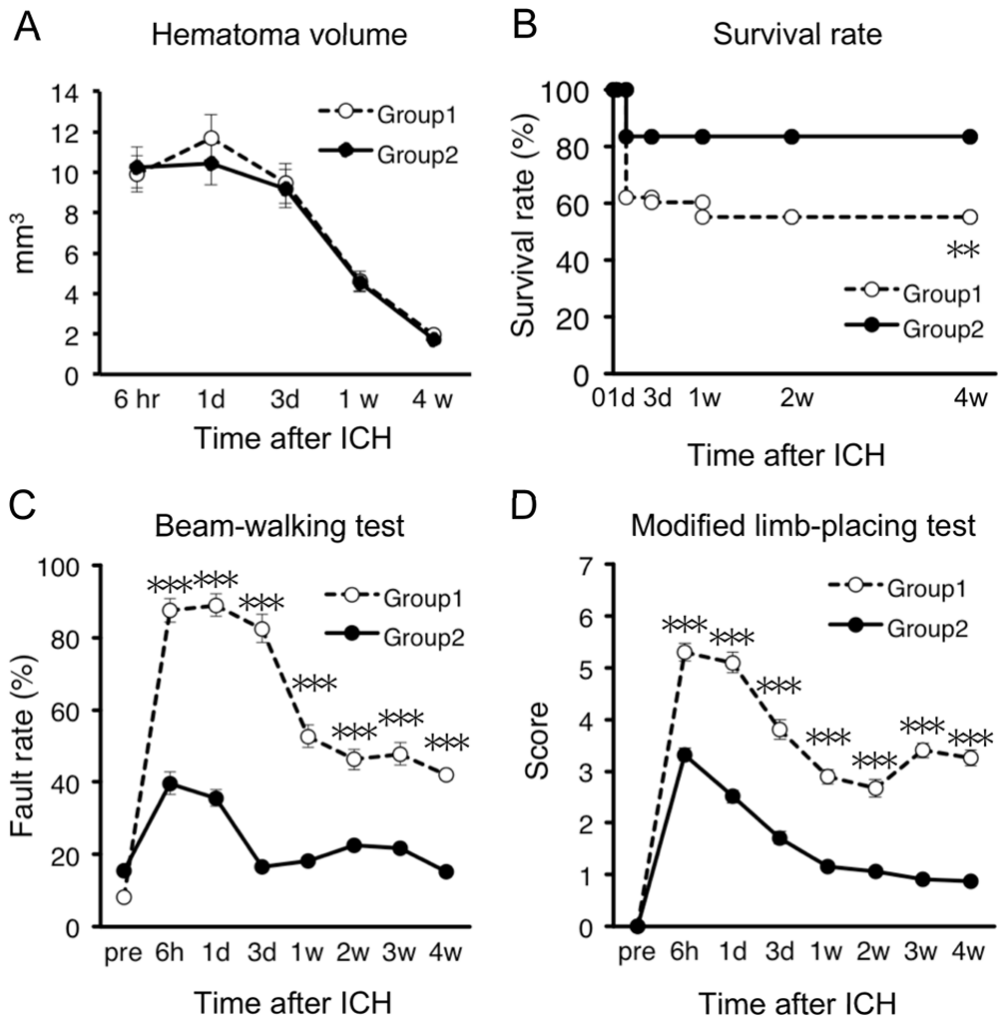
Induction of hemorrhage near IC resulted in high mortality rate and severe neurological dysfunctions. Group 1 mice (*n* = 56) received collagenase injection near IC, whereas group 2 mice (*n* = 55) received collagenase injection at a distant site from IC. (A) Time-dependent changes of hematoma volume. (B) Survival rate of mice after ICH. (C) Performance of mice in the beam-walking test evaluated by hindlimb fault rate. (D) Performance scores in modified limb-placing test. Data sets consist of values derived from all mice surviving at each time point. **P<0.01, ***P<0.001 versus group 2.

As mentioned above, two different stereotaxic coordinates were used for collagenase injection. In the next analysis, these two groups were compared with each other in terms of hematoma volume, mortality rate and degree of neurological dysfunctions. As shown in Fig. 3A, hematoma volume was not different between group 1 mice that received collagenase injection near IC (*n* = 56) and group 2 mice that received injection at a site distant from IC (*n* = 55). In contrast, the survival rate of group 1 mice was significantly lower than that of group 2 mice (Fig. 3B). In addition, group 1 mice exhibited significantly poorer performance than group 2 mice in the beam-walking test (Fig. 3C) and the modified limb-placing test (Fig. 3D).

### Hemorrhage Expansion into Internal Capsule is a Critical Predictor of High Mortality Rate and Enduring Neurological Dysfunctions

**Figure 4:**
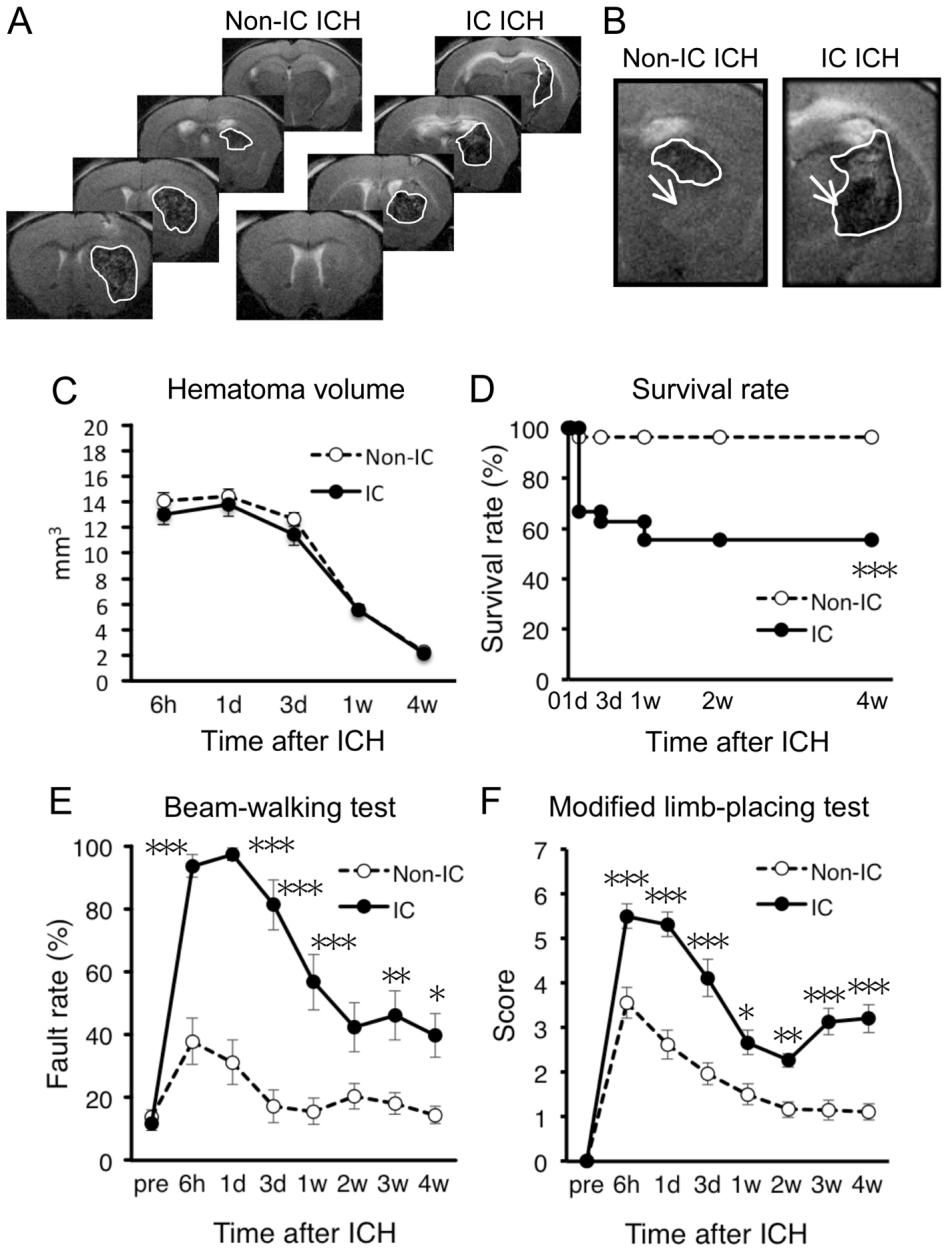
Invasion of hematoma into IC worsens mortality and neurological dysfunction. (A) Representative images of T2-MRI scans (+1.1, +0.1, −0.9 and −1.9 mm relative to bregma) in a non-IC ICH mouse and an IC ICH mouse at 6 h after induction of ICH. Boundary between hematoma and surrounding tissues is indicated by solid white line. (B) Close-up view of MRI images showing invasion of hematoma into IC in an IC ICH mouse. Arrows indicate the position of IC. (C–F) IC ICH mice (*n* = 26 at 6 h) and non-IC ICH mice (*n* = 27 at 6 h) were compared by hematoma volume (C), survival rate (D), hindlimb fault rate in the beam-walking test (E) and performance scores in the modified limb-placing test (F). Data sets consist of values derived from all mice surviving at each time point. *P<0.05, **P<0.01, ***P<0.001 versus non-IC ICH group.

Next we focused on relationship between the location of hematoma and the severity of symptoms. In the following series of experiments, only data from mice with hemorrhage larger than 5 mm^3^ were analyzed. IC has been known as a key site of lesion directly linked to motor hemiparesis after ICH in humans [10]–[12], and our results shown in Fig. 3 also demonstrated that hemorrhage induction near IC was associated with poor prognosis. To address whether invasion of IC by hematoma was indeed associated with high mortality rate and severity of neurological dysfunction in mouse ICH model, we divided mice into two subgroups based on the presence and the absence of hematoma expansion into IC (IC ICH and non-IC ICH, respectively; Fig. 4A and B). Mice with hemorrhage expansion into the lateral ventricle (LV) were excluded from this analysis. Hematoma volume of IC ICH mice (*n* = 26) and non-IC ICH mice (*n* = 27) was not different from each other (Fig. 4C). However, IC ICH mice exhibited significantly higher mortality rate than non-IC ICH mice (Fig. 4D). Moreover, IC ICH mice exhibited markedly impaired motor performance as compared to non-IC ICH mice in both beam-walking test (Fig. 4E) and modified limb-placing test (Fig. 4F).

**Figure 5:**
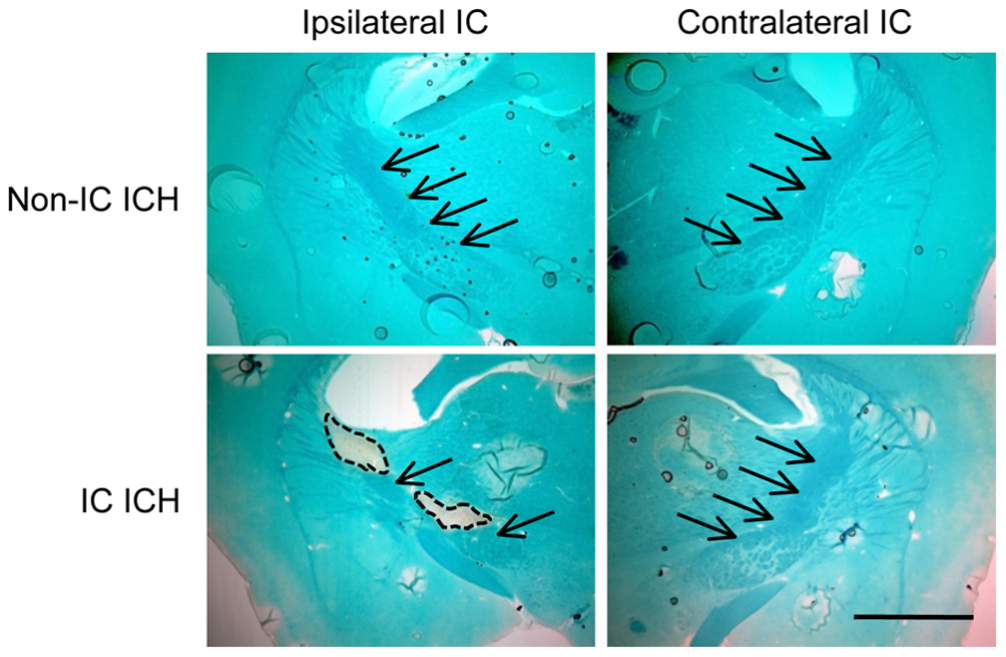
Axonal damage associated with invasion of hematoma into IC. Shown are representative images of Luxol fast blue staining of coronal sections (−1.4 mm relative to bregma) obtained from a non-IC ICH mouse and an IC ICH mouse at 4 weeks after induction of ICH. Arrows indicate IC, and broken lines indicate areas damaged by hematoma. Scale bar = 1 mm.

To confirm that hemorrhage induced axonal damage in IC ICH mice, we conducted Luxol fast blue staining for myelin at 4 weeks after induction of ICH. Damage of axon tracts was clearly observed as loss of staining of IC only in IC ICH mice (Fig. 5).

### Expansion of Hemorrhage into Lateral Ventricle is a Predictor of High Mortality Rate and Early Neurological Dysfunctions

**Figure 6:**
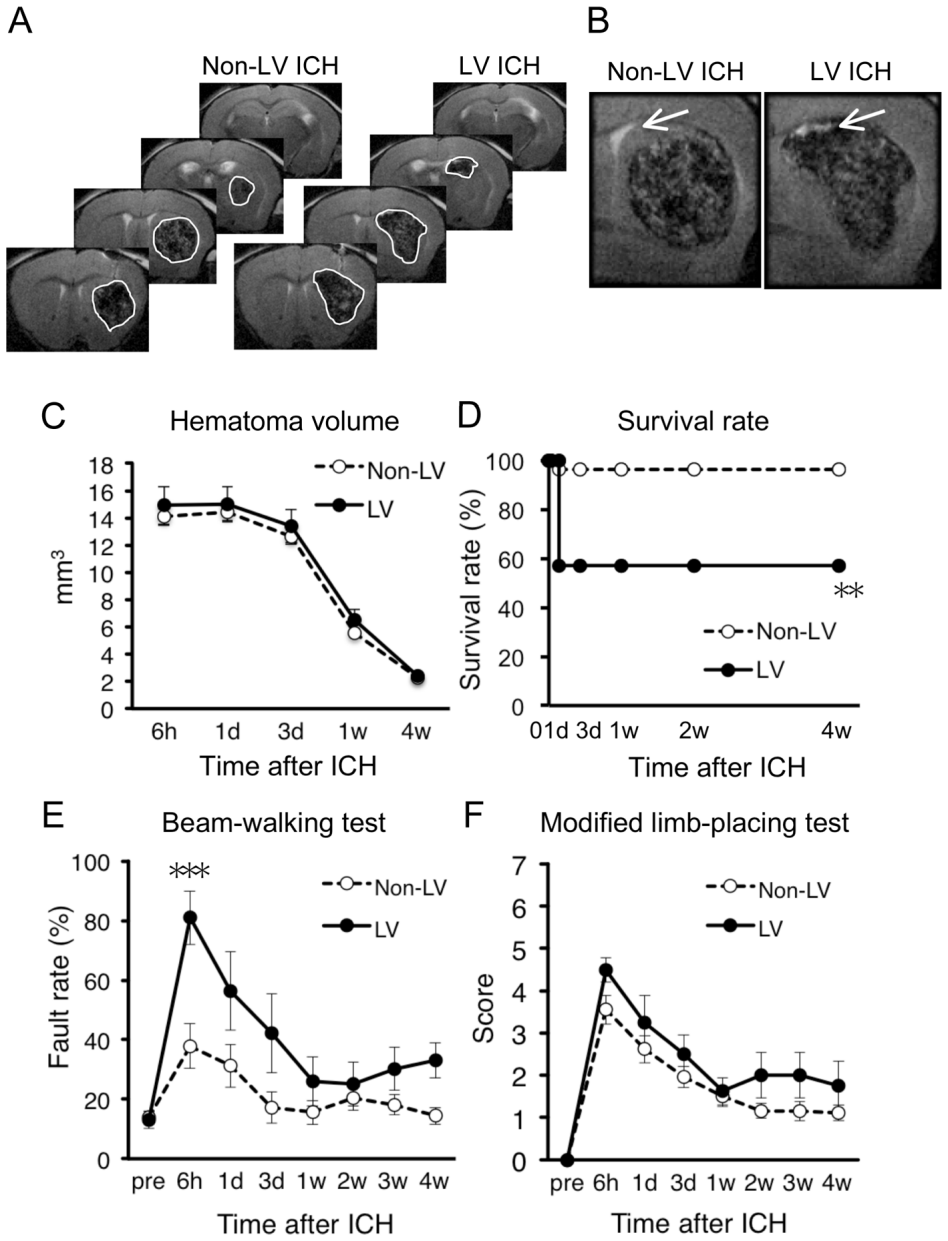
Invasion of hematoma into LV worsens mortality and initial neurological dysfunction. (A) Representative images of T2-MRI scans (+1.1, +0.1, −0.9 and −1.9 mm relative to bregma) in a non-LV ICH mouse and an LV ICH mouse at 6 h after induction of ICH. Boundary between hematoma and surrounding tissues is indicated by solid white line. (B) Close-up view of MRI images showing invasion of hematoma into LV in an LV ICH mouse. Arrows indicate LV. (C–F) LV ICH mice (*n* = 14 at 6 h) and non-LV ICH mice (*n* = 27 at 6 h) were compared by hematoma volume (C), survival rate (D), hindlimb fault rate in the beam-walking test (E) and performance score in the modified limb-placing test (F). Data sets consist of values derived from all mice surviving at each time point. ** *P*<0.01, *** *P*<0.001 versus non-LV ICH group.

In human ICH patients, expansion of hemorrhage into ventricles is associated with high mortality rate [6], [7] and severe neurological dysfunctions [7], [9]. In the next set of analysis, mice were divided into two groups based on the presence (LV ICH) and the absence (non-LV ICH) of hemorrhage expansion into LV (Fig. 6A and B). Mice with hemorrhage expansion into IC were excluded from this analysis. Although hematoma volume of non-LV ICH mice (*n* = 27) and that of LV ICH mice (*n* = 14) showed no significant difference during the entire course of experiments (Fig. 6C), LV ICH mice exhibited higher mortality rate than non-LV ICH mice (Fig. 6D). Additionally, LV ICH mice showed poorer performance in the beam-walking test than non-LV ICH mice, especially at early time point of 6 h after ICH (Fig. 6E). Performance in modified limb-placing test was not significantly different between these two groups (Fig. 6F).

### Hemorrhage Expansion into Base of the Brain does not Worsen Mortality Rate and Neurological Dysfunctions

**Figure 7:**
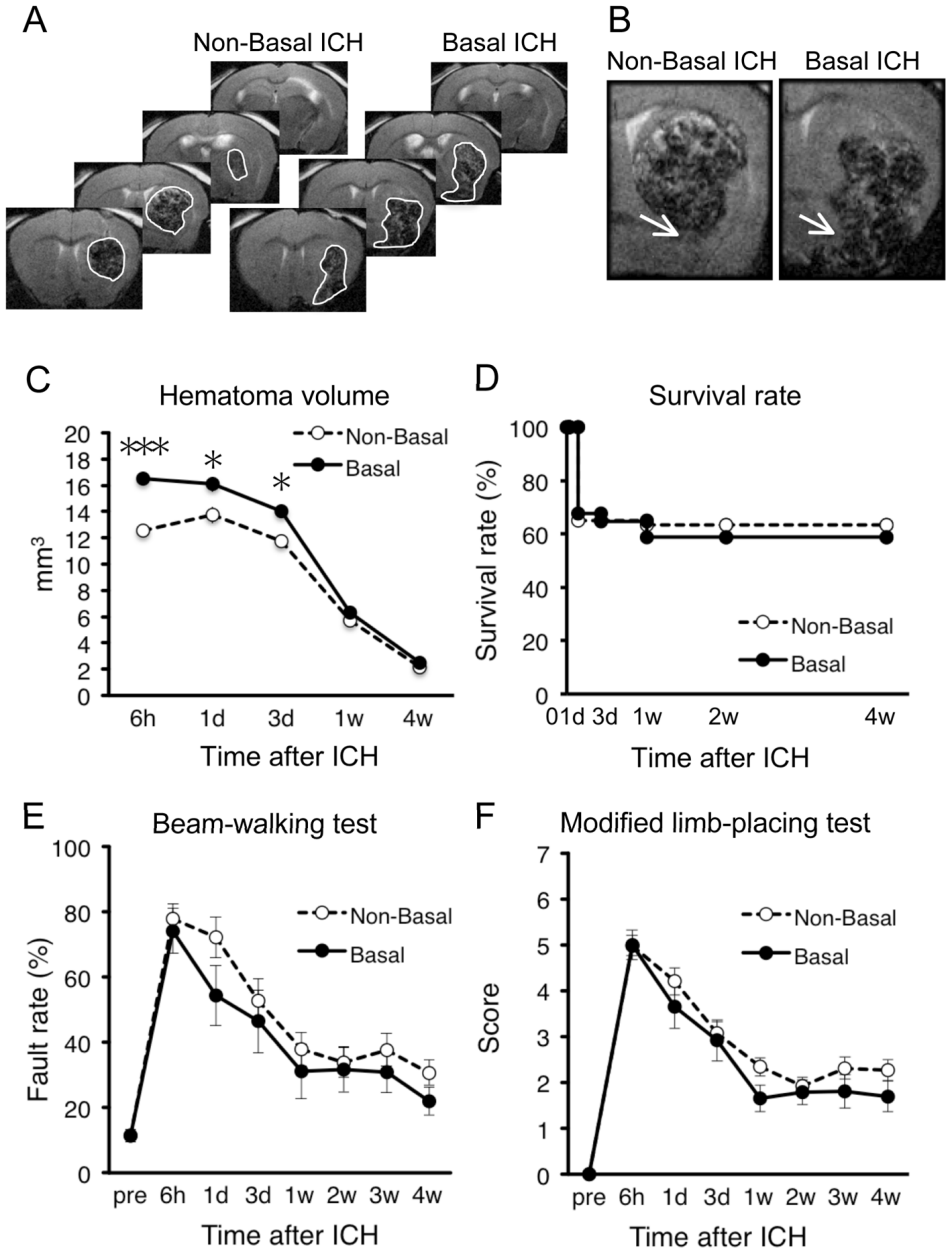
Invasion of hematoma into base of the brain does not affect mortality and neurological dysfunction. (A) Representative images of T2-MRI scans (+1.1, +0.1, −0.9 and −1.9 mm relative to bregma) in a non-Basal ICH mouse and a Basal ICH mouse at 6 h after induction of ICH. Boundary between hematoma and surrounding tissues is indicated by solid white line. (B) Close-up view of MRI images showing invasion of hematoma into the base of the brain in a Basal ICH mouse. Arrows indicate the base of the brain. (C–F) Basal ICH mice (*n* = 34 at 6 h) and non-Basal ICH mice (*n* = 59 at 6 h) were compared by hematoma volume (C), survival rate (D), hindlimb fault rate in the beam-walking test (E) and performance scores in the modified limb-placing test (F). Data sets consist of values derived from all mice surviving at each time point. * *P*<0.05, *** *P*<0.001 versus non-Basal ICH group.

In our ICH model, hemorrhage sometimes extended into the base of the brain (Fig. 7A and B). To address whether this unexpected expansion of hemorrhage affected neurological outcomes, we divided mice into two groups based on the presence and the absence of hematoma expansion into the base of the brain (Basal ICH and non-Basal ICH, respectively). Basal ICH was judged by expansion of hematoma into brain regions located ventral to the caudate-putamen, and affected regions included olfactory tubercle, magnocellular preoptic nucleus, piriform cortex and anterior portion of amygdaloid complex. In this analysis, mice with hemorrhage expansion into LV or IC were included. Hematoma volume in Basal ICH mice (*n* = 34) was significantly larger than that in non-Basal ICH mice (*n* = 59) at early time points (Fig. 7C), reflecting that large hematoma tended to spill over the caudate-putamen. In spite of the difference in hematoma size, mortality rate of Basal ICH mice and non-Basal ICH mice was virtually identical (Fig. 7D). In addition, performance scores in beam-walking test and modified limb-placing test were not different between Basal ICH mice and non-Basal ICH mice (Fig. 7E and F).

## Discussion

The present study addressed relationship between the properties of hemorrhage and the degree of prognosis after induction of striatal ICH in mice. The principal findings were that expansion of hematoma into either IC or LV, rather than the volume of hemorrhage per se, is a critical determinant of the mortality rate and the severity of neurological dysfunctions.

In human ICH patients, larger volume of hematoma has been associated with high mortality rate [5]. Here we demonstrated that this principle could also be applied in part to mouse ICH model. That is, all mice with hemorrhage smaller than 5 mm^3^ survived 4 weeks after induction of ICH, whereas mice with hemorrhage larger than 5 mm^3^ exhibited decreased survival rate. On the other hand, once the volume of hemorrhage exceeded 5 mm^3^, the mortality rate of mice seemed to be independent of the absolute volume of hemorrhage. This was also the case with the degree of sensorimotor dysfunctions that did not show significant difference among three groups with hemorrhage larger than 5 mm^3^. These results suggest that the volume of hemorrhage per se is not a good predictor of poor prognosis after ICH in mice, which is inconsistent with the observations in humans [5]. Causes of these apparent differences between mice and humans are unclear, but potential explanations are that three-dimensional architecture of the brain is largely different between mice and humans, and consequently, regions and structures affected by a given size of hemorrhage may well be different. These considerations also lead to the notion that specific regions in the brain may be a critical determinant of poor prognosis after ICH.

In this context, IC has been known as a region associated with poor outcome after ICH in human patients [10]–[12]. Several studies on animal models of ICH have also implicated IC injury in severe neurological dysfunctions. For example, Masuda et al. [19] have shown that small hemorrhage induced by collagenase injection near IC was sufficient to cause robust disability in motor functions. Our results in the present study are also consistent with the idea that hemorrhage expansion into IC results in poorer prognosis than hemorrhage without IC injury. Because IC is a white matter region that contains bundles of descending and ascending cortico-spinal tracts, it is reasonable to assume that IC injury by hemorrhage expansion substantially disrupts neural control of sensorimotor functions. On the other hand, precise reasons for increased mortality rate associated with IC-ICH are unclear, but potential causes may be related to the fact that IC contains not only cortico-spinal tracts but also cortico-bulbar tracts, part of which connects with vagus nerves that may mediate neurogenic shock in response to ICH. In the present study we could obtain IC injury nearly consistently when we made collagenase injection into the site near IC, and MRI analysis could easily validate the conditions of hemorrhage with respect to invasion of IC. Therefore, the present results indicate that ICH-associated IC injury model can be prepared in a practically reproducible manner in mice, which should promote preclinical investigations on pathophysiological mechanisms and therapeutic interventions against ICH-associated severe neurological dysfunctions.

We also demonstrated here that expansion of hemorrhage into LV resulted in increased mortality rate and exacerbated neurological dysfunctions. This observation corresponds well with those in human patients, where LV ICH has been associated with poor prognosis [6], [7], [9]. Notably, worsened outcome in LV ICH mice with regard to sensorimotor dysfunctions was observed only in the beam-walking test and was significant only at acute phase after ICH, which was substantially milder than that observed in IC ICH mice. These results imply that dysfunctions caused by LV ICH is related to exacerbation of transient pathological changes such as global hypoperfusion and edema, rather than exacerbation of persistent structural disturbances such as damages in axonal projections. This view on acutely exacerbated pathological events in LV ICH is consistent with the facts that the decreased survival rate of LV ICH mice was fully attributable to acute death within 24 h after ICH induction, and that mortality was closely associated with lower levels in motor performance at early time point.

In contrast to the results on IC ICH and LV ICH, whether or not hemorrhage expanded into the base of the brain was found to be irrelevant to the degree of prognosis. This observation may be causally related to the fact that the base of the brain does not contain regions critical for regulation of sensorimotor functions assessed in the present study. The results also add further evidence that the absolute volume of hemorrhage is not a major factor determining the extent of prognosis.

With a few exceptions [15], [19], details of the relationship between the properties of hemorrhage and the degree of neurological outcome have not been addressed in animal models of ICH. The study by MacLellan et al. [15] demonstrated correlation between the degree of neurological dysfunctions and the size of injury in a retrospective manner, based on the volume of lost tissue at 30 d after ICH. In contrast, our present results indicate that the mortality of animals and the degree of neurological dysfunctions can be predicted by MRI inspections of the conditions of hemorrhage at an early time point. These prospective procedures are valuable in utilizing the animal model for examination of neuropathological mechanisms and also for accurate evaluation of the effects of potential neuroprotective drug therapies.
